# Public Interest in Acne on the Internet: Comparison of Search Information From Google Trends and Naver

**DOI:** 10.2196/19427

**Published:** 2020-10-26

**Authors:** Tae Heum Park, Woo Il Kim, Suyeon Park, Jaeouk Ahn, Moon Kyun Cho, Sooyoung Kim

**Affiliations:** 1 Department of Dermatology Soonchunhyang University Hospital Seoul Republic of Korea; 2 Department of Biostatistics Soonchunhyang University Hospital Seoul Republic of Korea; 3 Department of Medical IT Engineering Soonchunhyang University Asan Republic of Korea

**Keywords:** acne vulgaris, internet, infodemiology, infoveillance, cosmetics, diet, dermatology, Google

## Abstract

**Background:**

Acne vulgaris is a common skin disease primarily affecting young adults. Given that the internet has become a major source of health information, especially among the young, the internet is a powerful tool of communication and has a significant influence on patients.

**Objective:**

This study aimed to clarify the features of patients’ interest in and evaluate the quality of information about acne vulgaris on the internet.

**Methods:**

We compared the search volumes on acne vulgaris with those of other dermatological diseases using Google Trends from January 2004 to August 2019. We also determined the search volumes for relevant keywords of acne vulgaris on Google and Naver and evaluated the quality of answers to the queries in KnowledgeiN.

**Results:**

The regression analysis of Google Trends data demonstrated that the patients’ interest in acne vulgaris was higher than that for other dermatological diseases, such as atopic dermatitis (β=−20.33, 95% CI –22.27 to –18.39, *P*<.001) and urticaria (β=−27.09, 95% CI –29.03 to –25.15, *P*<.001) and has increased yearly (β=2.38, 95% CI 2.05 to 2.71, *P*<.001). The search volume for acne vulgaris was significantly higher in the summer than in the spring (β=–5.04, 95% CI –9.21 to –0.88, *P*=.018) and on weekends than on weekdays (β=–6.68, 95% CI –13.18 to –0.18, *P*=.044). The most frequently searched relevant keywords with “acne vulgaris” and “cause” were “stress,” “food,” and “cosmetics.” Among food, the 2 highest acne vulgaris–related keywords were milk and wheat in Naver and coffee and ramen in Google. The queries in Naver KnowledgeiN were mostly answered by a Korean traditional medicine doctor (53.4%) or the public (33.6%), but only 12.0% by dermatologists.

**Conclusions:**

Physicians should be aware of patients’ interest in and beliefs about acne vulgaris to provide the best patient education and care, both online and in the clinic.

## Introduction

### Background

The internet has spread widely since the 1980s and has become a powerful tool and route of communication. People increasingly use it to find information about health problems. According to the *Measuring the Information Society Report* from 2018 by the International Telecommunication Union, the internet usage rate in South Korea was 95.10% in 2018 [1]. According to 2018 internet usage statistics by the Ministry of Science and Information and Communications Technology of South Korea, the internet usage rate was 91.5% (46,124,694 persons), and the average daily internet usage time in South Korea was 2 hours 15 minutes [2]. As internet usage increases, the influence of the internet is also growing. According to the 2018 internet usage statistics by the Ministry of Science and Information and Communications Technology of South Korea, we use it for viewing movies, videos, and images (87.0%); shopping (62.0%); banking (63.7%); and communication through messengers (95.9%) [2]. In the face of the rapid expansion of smartphones, smartphones have made the internet more ubiquitous; we can get any information wherever we are, which contributes to the percentage of people using the mobile internet (90.4%) in 2018 in South Korea. In particular, young people actively use the internet to exchange their thoughts and get information, including medical and health care information. Among people aged in their 10s, 20s, and 30s, the ratios of people using the internet were 99.9%, 99.9%, and 99.9%, respectively, and those of people using the mobile internet were 98.7%, 99.9%, and 99.9%, respectively [2].

Acne vulgaris is a very common dermatological disease and the 8th most common disease worldwide; it is estimated to affect 633 million people globally [3,4]. It primarily affects young adults, mostly in their teens, 20s, and 30s [5]. Four mechanisms are considered important in the pathogenesis of acne vulgaris: (1) sebaceous hypersecretion, (2) hair follicle hyperkeratosis, (3) colonization of *Cutibacterium acnes*, and (4) inflammatory reactions [6]. External factors such as the environment are also known as important risk factors. Mechanical stimulation like friction, psychological stress [7], and excessive sweating have also been suggested as causes of acne vulgaris. Food is also a possible cause, but the relationship is still unclear, even though there are studies that show a relationship between acne vulgaris and food. The most commonly involved site of acne vulgaris is the face, followed by the neck, back, and chest. Clinically, various kinds of skin lesions are possible: comedon, papule, pustule, and nodule. Deep skin lesions can result in scars, and it is estimated that 95% of acne vulgaris patients have acne scars [8], which can induce serious psychosocial problems [4].

### Goal of This Study

Acne vulgaris is prevalent. It is observed in 80%-90% of adolescents and young adults, and it has high social interest because of its cosmetic sequelae and psychosocial problems. The purpose of this study was to clarify the features of patients’ interest in and evaluate the quality of information about acne vulgaris on the internet. Therefore, we aimed to investigate public interest in acne vulgaris by assessing the search volume of acne vulgaris–related keywords through the major search sites (Naver, Google) that are widely used in South Korea. In addition, we analyzed who answered questions about acne vulgaris on the internet and evaluated the answers posted on internet sites to investigate the accuracy of the information on the internet for public health.

## Methods

### Study Design

We used the search portal sites Naver and Google, which are among the most commonly used search sites in South Korea.

First, we compared the interest in acne vulgaris and other dermatological diseases by using Google Trends, a website that analyzes the popularity of top search queries in Google Search across various regions and languages. Google Trends provides a search volume index (0 to 100), which is the search volume relative to the total number of searches performed on Google. We compared the public interest in acne vulgaris to that of other dermatological disorders, such as atopic dermatitis or urticaria, as well as the yearly and seasonal trends in interest, using regression analysis of 15 years’ worth of Google Trends data. From the regression analysis of Google Trends 90-day data, we looked at whether there is a difference between interest on weekends and on weekdays. To determine whether there is a correlation between Google Trends geographic data and personal income, private consumption, or composition of the population, we performed correlation analyses. Keywords used for the search were “acne vulgaris,” “urticaria” (urticae or urtica were not included), and “atopic dermatitis” (atopic eczema was also included) in Korean.

Next, we compared search volumes using keywords for “acne vulgaris, treatment,” “acne vulgaris, laser,” “acne vulgaris, cause,” “acne vulgaris, diet,” and “acne vulgaris, cosmetics” in Korean in Google and Naver KnowledgeiN. We also sorted 500 answers by responders (dermatologist, general practitioner or specialist medical doctor, Korean medicine doctor, or general public) in Naver KnowledgeiN.

### Statistical Analysis

We used linear regression analysis to observe yearly trends and seasonal trends; correlation analysis to determine correlations between Google Trends geographic data and personal income, private consumption, or composition of population; and text mining to express the search volume of acne vulgaris–related keywords. The statistics program R 3.6.2 was used to analyze data, and *P*<.05 was considered statistically significant.

## Results

### Acne Vulgaris is One of the Most Popular Dermatological Diseases on the Internet

**Figure 1:**
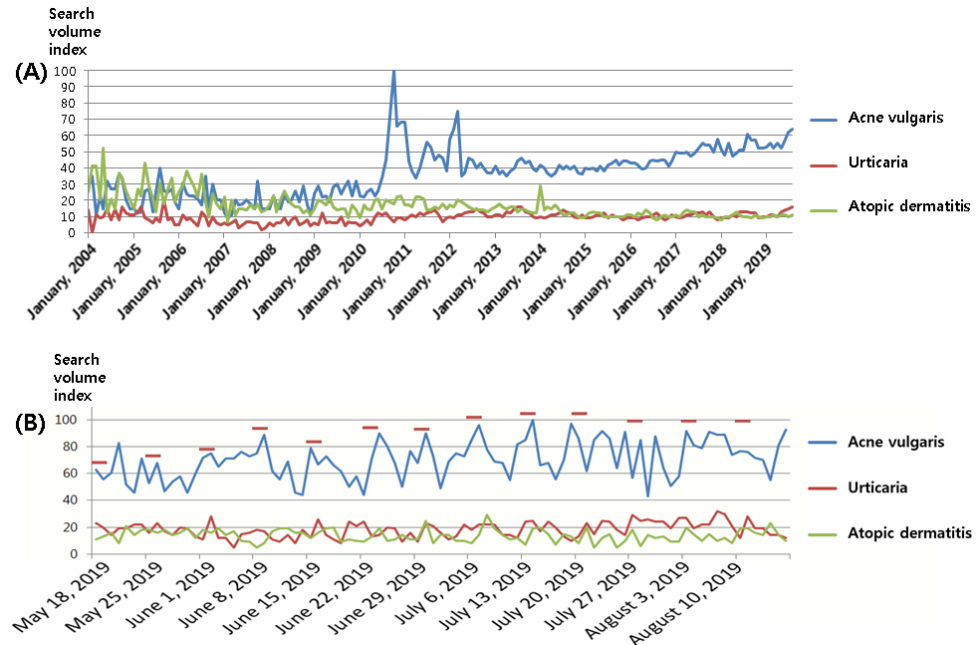
Temporal trends in searches for acne: (A) Google Trends time data (15 years). The search volume index (0 to 100) represents the search volumes relative to the total number of Google searches. (B) Google Trends time data (90 days). The horizontal bars indicate weekends.

The regression analysis of Google Trends data for 15 years demonstrated that patients’ interest in acne vulgaris was higher than the interest in atopic dermatitis (β=–20.33, 95% CI –22.27 to –18.39, *P*<.001) and urticaria (β=–27.09*,* 95% CI –29.03 to –25.15, *P*<.001). Public interest in acne vulgaris has increased yearly (β=2.38, 95% CI 2.05 to 2.71, *P*<.001) and was higher in summer than in spring (β=–5.04, 95% CI –9.21 to –0.88, *P*=.018). But there was no significant difference between other seasons. For the temporal trends between weekends and weekdays, the interest in acne vulgaris on weekends was higher than on weekdays (β=–6.68, 95% CI –13.18 to –0.18, *P*=.044; Figure 1).

### Seoul Has the Greatest Interest in Acne Vulgaris on the Internet Among Cities and Provinces

**Figure 2:**
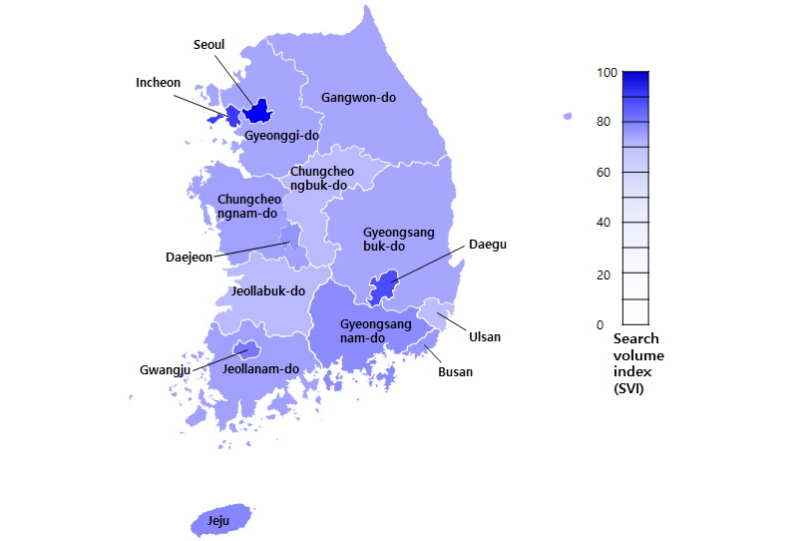
Regional trends in internet searches for acne.

We also determined interest in acne vulgaris among cities and provinces through Google Trends geographic data. Seoul had the highest interest in acne vulgaris among cities and provinces, followed by Incheon and Daegu. Jeollabuk-do and Chungcheongbuk-do had the lowest interest. There was no significant correlation between search volumes and personal income (correlation coefficient=0.30, *P*=.27) or between search volumes and private consumption (correlation coefficient=0.37, *P*=.16; Table 1; Figure 2). There was also no significant correlation between search volume and age (Table 2).

**Table 1:** Acne vulgaris search volume and personal income in cities and provinces.

Cities and provinces	Search volume	Personal income (annual, KRW)	Private consumption (annual, KRW)
Seoul	100	21,429,000	20,211,000
Incheon	91	17,550,000	14,486,000
Daegu	88	17,568,000	15,682,000
Gwangju	81	17,343,000	16,122,000
Jeju	79	17,464,000	15,107,000
Gyeongsangnam-do	78	16,864,000	14,735,000
Daejeon	77	18,454,000	16,286,000
Busan	76	18,332,000	16,208,000
Chungcheongnam-do	75	17,613,000	14,047,000
Jeollanam-do	75	15,938,000	14,112,000
Gyeonggi-do	74	18,580,000	15,786,000
Gyeongsangbuk-do	74	16,504,000	14,395,000
Gangwon-do	74	16,583,000	14,957,000
Ulsan	73	19,912,000	16,494,000
Jeollabuk-do	71	16,848,000	14,194,000
Chungcheongbuk-do	71	17,030,000	14,381,000

**Table 2:** Acne vulgaris search volume and population composition in cities and provinces.

Cities and provinces	Search volume			Ratio of the population in the age range, %					
	2018	2017	2016	10s to 20s (2018)	10s (2018)	10s to 20s (2017)	10s (2017)	10s to 20s (2016)	10s (2016)
Seoul	100	100	89	23.60	8.76	23.76	9.08	23.94	9.41
Incheon	83	81	95	23.76	9.89	24.13	10.23	24.40	10.58
Daegu	88	80	75	23.58	10.14	24.00	10.58	24.31	11.03
Gwangju	73	82	69	25.88	11.78	26.19	12.28	26.48	12.77
Jeju	90	78	100	23.30	11.05	23.53	11.39	23.74	11.81
Gyeongsangnam-do	77	80	80	21.93	10.25	22.31	10.56	22.68	10.90
Daejeon	82	69	99	25.13	10.77	25.43	11.18	25.67	11.62
Busan	73	70	65	21.53	8.61	21.95	8.93	22.31	9.30
Chungcheongnam-do	70	70	88	21.73	10.04	22.05	10.32	22.35	10.61
Jeollanam-do	76	76	81	20.73	9.75	21.04	10.11	21.34	10.50
Gyeonggi-do	77	72	76	23.97	10.59	24.33	10.96	24.59	11.32
Gyeongsangbuk-do	81	69	71	20.37	9.16	20.80	9.45	21.22	9.80
Gangwon-do	80	66	73	21.58	9.72	22.12	10.12	22.46	10.54
Ulsan	63	73	68	23.79	10.52	24.36	10.89	24.86	11.32
Jeollabuk-do	74	64	72	22.23	10.39	22.64	10.78	22.90	11.13
Chungcheongbuk-do	85	73	58	22.55	10.27	22.92	10.40	23.24	10.79

### Most Frequently Searched Relevant Keywords Were Cosmetics, Food, Laser, and Hormone

We searched Naver KnowledgeiN and Google for topics combined with “acne vulgaris” in 5 areas: treatment, cause, cosmetics, laser, and food (Table 3).

When comparing the number of search results from Naver KnowledgeiN with that from Google, Google had a greater number of search results: “acne vulgaris and cause,” 1,078,537/12,797,237, 8.4%, Naver and 11,718,700/12,797,237, 91.6%, Google; “acne vulgaris and food,” 257,137/5,740,137, 4.5%, Naver and 5,483,000/5,740,137, 95.5%, Google; “acne vulgaris and cosmetics,” 1,250,879/14,097,879, 8.9%, Naver and 12,847,000/14,097,879, 91.1%, Google; “acne vulgaris and treatment,” 277,918/2,035,700, 13.7%, Naver and 1,757,782/2,035,700, 86.3%, Google; and “acne vulgaris and laser,” 159,158/1,908,158, 8.3%, Naver and 1,749,000/1,908,158, 91.7%, Google.

**Table 3:** Frequently searched relevant keywords determined by text mining.

Topic		Naver	Google
**Acne and cause**			
	Stress	371,735	1,520,000
	Food	265,991	1,890,000
	Cosmetics	29,249	6,630,000
**Acne and food**			
	Milk	57,135	478,000
	Flour	57,161	167,000
	Coffee	27,005	1,460,000
	Ramen	4285	1,370,000
**Acne and cosmetics**			
	Toner	334,502	6,020,000
	Lotion	332,153	814,000
	Cleansing foams	57,135	472,000
	Essences	68,667	1,250,000
**Acne and treatment**			
	Lasers	168,750	1,250,000
	Antibiotics	54,363	184,000
**Acne and laser**			
	Fraxel laser	40,542	154,000
	Photodynamic therapy	29,933	125,000
	Toning laser	18,613	428,000
	Intense pulsed light	16,379	280,000
	LED	1378	316,000

### Information via the Internet Has Been Provided Mostly by Nonexperts

We sorted 500 answers by responders in Naver KnowledgeiN. The queries in Naver KnowledgeiN were mostly answered by Korean traditional medical doctors (267/500, 53.4%), but only 12.0% (60/500) by dermatologists, 33.6% (168/500) by the public, and 1.0% (5/500) by a general practitioner or a specialist medical doctor. The most common question about acne vulgaris was treatment (393/500, 78.6%), followed by cause (61/500, 12.2%), cosmetics (22/500, 4.4%), and laser (9/500, 1.8%).

## Discussion

### Principal Findings

People have greater interest in acne vulgaris than in other dermatological disorders, and patients’ interest in acne vulgaris has significantly increased yearly. There were also significant differences in interest between cities and provinces. But there were no significant correlations between public interest and personal income, private consumption, or composition of the population. We selected acne-related topics that people would be curious about: cause, food, cosmetics, treatment, and laser. Among the foods, there were many searches for milk, wheat, and coffee. Regarding lasers, Fraxel laser, photodynamic therapy (Naver), toning laser, and LED (Google) were of high interest. Answers to the questions on the internet were mostly provided by Korean traditional medical doctors (53.4%) or the public without expertise (33.6%) and only 12.2% by dermatologists.

Public interest in beauty has been increasing in South Korea. According to the International Society of Aesthetic Plastic Surgery, the size of the Korean plastic surgery market was estimated at about 5 trillion won in 2017, which was about a quarter of the world market, and the annual number of plastic surgeries per 1000 people was estimated at 13.5, making it first in the world [9]. The market size of the cosmetics and plastic surgery industries in South Korea has been growing [9,10], which could explain why the interest in acne vulgaris is high and increased much more abruptly than other dermatological disorders, such as atopic dermatitis. Another reason for this high interest may be that acne vulgaris mostly affects young people, who use the internet much more frequently.

People's lifestyles and thoughts are related to internet search volumes. Because acne vulgaris frequently affects young people who work or go to school on weekdays [5], the search volume for acne vulgaris was significantly higher on weekends than on weekdays. Conventionally, people might think that the higher their income, the more they care about their appearance, but there was no significant correlation between public interest and personal income or private consumption. From this, we can see that people's interest in beauty is universal regardless of economic condition. Related to this finding, some studies have revealed that concern about physical appearance has no association with socioeconomic status [11].

The internet can probably help to identify etiology and epidemiology of disease [12]. It is known that acne vulgaris tends to get worse in the summer [13,14]. High temperatures can change sebum excretion [15], which can worsen acne vulgaris. Consistently, we found that the search volume for acne vulgaris was significantly higher in summer than in spring. Several studies have suggested that dairy products, chocolate, and hyperglycemic foods, such as ramen and flour, can exacerbate acne vulgaris [16-22]. Consistent with these studies, we found that people frequently searched for ramen, flour, chocolate, or milk along with acne vulgaris. Stress and hormones, which were frequently searched by the public on the internet, are also well known to exacerbate acne vulgaris [7,23-25]. Similarly, there are many other studies attempting to estimate epidemiology or track outbreaks of various kinds of diseases, such as influenza, cellulitis, cancer, and tuberculosis, by using the internet [12,26,27].

The power of mass media is extremely strong, even in the medical field [28,29]. In Figure 1A, there was a peak in search volume around the second week of October 2010, probably caused by news articles reported on October 13, 2010, from Korean major broadcasters such as MBC, SBS, or MBN that emphasized the relevance of food and acne vulgaris. In addition to conventional mass media, the internet, smartphones, and social media are also emerging as influential tools [30-32]. In 2010, there was an abrupt spread of smartphones in South Korea, which enabled people to get health information wherever they are and contributed to the peak of search volumes in 2010. Physicians can use the powerful internet in the medical field [33]. First, through the internet, information about which the public is curious can be easily identified. Second, it can be a helpful tool for patient education because the internet is easily accessible and interactive and can provide media-based material, such as photos and videos. About this, especially in the field of skin cancer, several studies have shown that dermatologists can even effectively change people’s skin-related health behavior through an internet intervention and can thus reduce the prevalence of skin diseases [34-36]. Through internet-based deep learning, diagnostic tools for skin cancer are even currently under development [37,38].

However, there is also the probability that false information could be provided, which can also be a major health risk. Much of the information was provided by nonexperts, such as the public or Korean traditional medical doctors, rather than by experts, that is, dermatologists, so the risk is high that false information might spread and endanger patients. Moreover, during data collection, we found manipulation for the purpose of promotion; in Naver KnowledgeiN, someone posted the same questions and adopted only one user as a best answer user. In this way, some people, if they have dishonest intentions, can spread misleading information very easily through the internet. Newspapers and TV news reports also have reported cases of adverse health effects because of incorrect medical information on the internet (Table 4). The Naver online café, Anaki, has greatly restricted the use of medicines for children and has even held chicken pox parties, causing a great deal of controversy. In addition, in the United States, conspiracy theories about vaccination spread on the internet, and the measles vaccination rate dropped significantly, which caused another measles pandemic [39]. As such, in the medical field, the internet has both aspects, in that helpful information can be provided to patients and yet wrong information can be provided and cause serious harm to patients.

**Table 4:** Risk cases for dissemination of unverified facts.

Source or harm	Example
Anaki	The Naver Café, with a membership as high as 60,000 people, caused problems with the dissemination of unfounded medical information, such as neglecting children’s illness, refusing vaccinations, throwing chicken pox parties, and selling untested medicines.
Anyemo	This antivaccine movement website refused vaccinations without any reasonable evidence, which could cause pandemics of infectious diseases.
Measles epidemic in the United States	The measles epidemic in the United States has greatly increased because of the decrease in vaccination resulting from unfounded stories about vaccination spread through social media platforms such as Facebook.

### Limitations

There are several limitations to this study. First, Google Trends provides only a relative search volume index, not the absolute search volume, and does not provide a way to calculate the search volume index. Second, we could not find statistically significant factors affecting geographic differences. Personal income, personal consumption, and composition of the population had only weak positive correlations with acne vulgaris. Third, there are many advertisements about acne vulgaris, which might not represent the real interest of the public. Fourth, we could not analyze the exact accuracy of information, but only estimate its quality through the ratio of who answered the questions in Naver KnowledgeiN. Last, it is not an interventional study. Further interventional studies are needed to measure the real effect of the internet in the real world.

### Conclusions

As part of patient education, we dermatologists need to correct wrong information. In addition, we can use the internet for patient education by launching official websites that provide accurate information about skin diseases or by operating dermatologists’ Q&A sites. As such, we need to try both in and outside the office to understand people’s interests and beliefs in the internet space and try to intervene through the internet and smartphones to provide the best treatment and education for patients with skin diseases.
